# Logic Gates Based on Skyrmions

**DOI:** 10.3390/nano16020135

**Published:** 2026-01-19

**Authors:** Yun Shu, Qianrui Li, Wei Zhang, Yi Peng, Ping Lai, Guoping Zhao

**Affiliations:** 1 School of Physics and Electronic and Electrical Engineering, Aba Teachers College, Wenchuan 623002, China; 2 State Key Laboratory of Spintronics, Hangzhou International Innovation Institute, Beihang University, Hangzhou 311115, China; zhangwei01@buaa.edu.cn (W.Z.);; 3 School of Integrated Circuit Science and Engineering, Beihang University, Beijing 100191, China; 4 College of Physics and Electronic Engineering, Sichuan Normal University, Chengdu 610068, China

**Keywords:** logic gates, magnetic skyrmions, CMOS, ultra-low-power

## Abstract

Traditional complementary metal-oxide-semiconductor (CMOS) logic gates serve as the fundamental building blocks of modern computing, operating through the electron charge manipulation wherein binary information is encoded as distinct high- and low-voltage states. However, as physical dimensions approach the quantum limit, conventional logic gates encounter fundamental bottlenecks, including power consumption barriers, memory limitations, and a significant increase in static power dissipation. Consequently, the pursuit of novel low-power computing methodologies has emerged as a research hotspot in the post-Moore era. Logic gates based on magnetic skyrmions constitute a highly promising candidate in this context. Magnetic skyrmions, nanoscale quasiparticles endowed with topological protection, offer ideal carriers for information transmission due to their exceptional stability and mobility. In this work, we provide a concise overview of the current development status and underlying operating principles of magnetic skyrmion logic gates across various magnetic materials, including ferromagnetic, synthetic antiferromagnetic, and antiferromagnetic systems. The introduction of magnetic skyrmion-based logical operations represents a paradigm shift from traditional Boolean logic to architectures integrating memory and computation, as well as brain-inspired neuromorphic computing. Although significant challenges remain in the synthesis of materials, fabrication, and detection, magnetic skyrmion-based logic computing holds considerable potential as a future ultra-low-power computing technology.

## 1. Introduction

Intrinsic limitations have constrained traditional logic gates near their physical boundaries, prompting intense research into higher-performance logic computing methodologies. Among the emerging technologies, magnetic skyrmion–based logic computing has naturally gained attention. Owing to their nanoscale dimensions, high stability and mobility, magnetic skyrmions have become a focal point of current research. Originally proposed by British physicist Tony Skyrme in 1962 as a topological soliton model in nuclear physics [1], skyrmions were first observed in B20 ferromagnetic (FM) materials via neutron scattering in 2009 [2], followed by direct real-space observation in 2010 [3]. Moreover, various derivative structures of skyrmions have been systematically characterized and classified, such as antiskyrmions [4,5,6,7,8,9,10,11], bimerons [12,13,14,15,16,17], skyrmioniums [18,19,20,21,22,23,24,25,26,27], biskyrmions [28,29,30], half skyrmions [31,32,33,34], hopfions [35,36,37,38,39,40] and skyrmion bundles [41,42]. Consequently, the stable generation [43,44,45,46,47,48,49,50,51,52,53,54], dynamic characterization [55,56,57,58,59,60,61,62,63,64,65], and detection methodologies [66,67,68,69,70,71,72] of magnetic skyrmions have emerged as central research topics. Recent studies have demonstrated that magnetic skyrmions can persist stably in synthetic antiferromagnetic (SAFM) [73,74,75,76,77,78,79,80,81] and antiferromagnetic (AFM) materials [82,83,84,85,86], in which the skyrmion Hall effect (SkHE) is offset, making them highly suitable for information storage applications. In addition, diverse types of skyrmions are stable in ferrimagnets [87,88,89], frustrated magnets [90,91,92,93,94], ferroelectrics [95,96,97], semiconductors [98], superconductors [99] and two-dimensional (2D) systems [100]. Furthermore, magnetic skyrmions can be effectively manipulated using electric currents [101,102,103], electric fields [104,105,106,107], magnetic fields [108,109,110,111,112,113,114], spin waves [88,115,116,117,118,119], lasers [118,119,120,121,122,123], ion irradiation [124,125,126], strain [127,128], microwave field [129], and surface acoustic waves [130]. Notably, the reliable generation of single skyrmions is crucial for the realization of skyrmion-based logic architectures. For instance, Romming et al. employ localized spin-polarized currents from a scanning tunneling microscope to controllably write and delete individual skyrmions in a PdFe bilayer on Ir(111) at 8 K [50]. Similarly, Woo et al. successfully generate and annihilate isolated magnetic skyrmions in a FM GdFeCo film at room temperature using time-resolved X-ray pump-probe measurements [49]. These findings provide a solid foundation for future technological applications of magnetic skyrmions [131,132,133,134,135,136,137,138,139,140].

**Figure 1:**
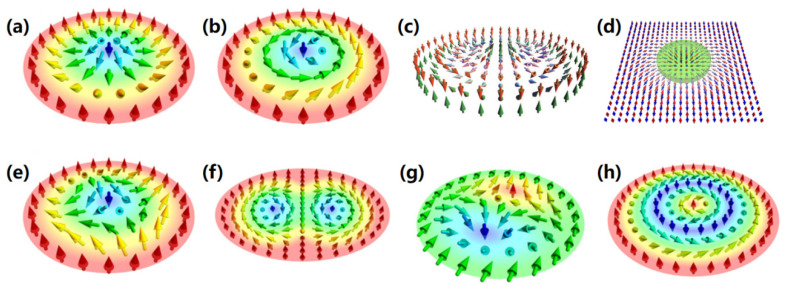
Various magnetic skyrmions and their counterparts. (**a**) Néel-type skyrmion [136]. (**b**) Bloch-type skyrmion [136]. (**c**) Synthetic antiferromagnetic skyrmion [140]. (**d**) Antiferromagnetic skyrmion [141]. (**e**) Antiskyrmion [136]. (**f**) Biskyrmion [136]. (**g**) Bimeron [136]. (**h**) Skyrmionium [136].

Figure 1 illustrates magnetic skyrmions and their corresponding counterparts [136,140,141].

Given the quasiparticle characteristics and rich dynamic behaviors of magnetic skyrmions, a variety of spintronic devices based on these have been proposed, including racetrack memories [57,142,143,144,145,146], diodes [147,148,149,150,151], nano-oscillators [152,153,154,155,156,157,158,159,160,161,162,163], neurons [164,165,166,167,168,169,170,171,172], and logic gates [77,141,173,174,175,176,177,178,179,180,181,182,183,184], among others. Notably, compared to other devices, significantly more studies have focused on magnetic skyrmion-based logic gates. Since the first proposal of a magnetic skyrmion logic gate in 2015 [173], research in this area has steadily increased, with over one hundred related studies published to date. Between 2020 and 2023, our team proposed and realized logic gates based on FM, SAFM, and AFM skyrmions [77,141,183,184]. These efforts provide a practical foundation for selecting suitable skyrmion materials and offer essential guidance for formulating design principles and optimizing future skyrmion-based logic devices. Accordingly, this article reviews the operating principles and implementations of magnetic skyrmion-based logic gates in various materials. It provides a comparative analysis of their potential advantages over conventional logic gates with respect to energy consumption, non-volatility, and expansibility, as well as discusses their prospects for enabling low-power computing in the post-Moore’s Law era.

## 2. The Logic Gates in Ferromagnetic Materials

**Figure 2:**
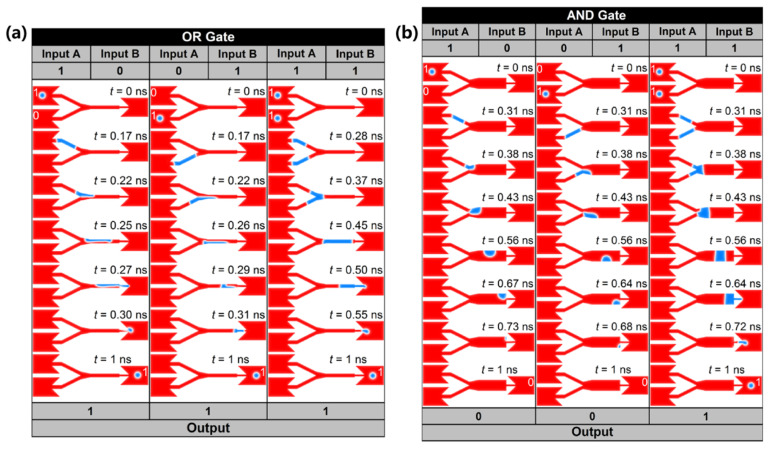
Schematic diagrams illustrate the fundamental principles of logic gates based on magnetic skyrmions [173]. (**a**) The logic OR gate; (**b**) The logic AND gate.

Studies show that the reversible transitions can occur between magnetic domain walls and magnetic skyrmions within specific racetracks [185]. In 2015, Zhang leverages this property to innovatively propose the first magnetic skyrmion-based logic gates in 2015 [173], as illustrated in Figure 2.

The primary structure of this logic gate consists of a Y-shaped nano-racetrack that connects two inputs and a single output. Figure 2a displays the operation of the OR gate, whose fundamental operations include “0 + 0 = 0”, “1 + 0 = 1”, “0 + 1 = 1”, and “1 + 1 = 1”. In this configuration, the presence of magnetic skyrmions denotes a logical signal of “1”, while their absence represents a logical signal of “0”, such that “0 + 0 = 0” indicates that neither an input nor an output is present. In the left column of Figure 2a, when a magnetic skyrmion is generated only at input A, representing the input signal is “1 + 0”. Driven by the electric current, the skyrmion at input A enters the narrow nano-racetrack from the wider part, and then transforms into a magnetic domain wall and propagates through the narrow channel. Eventually, it converts back into a skyrmion in the wide nano-racetrack at the output, thereby achieving the operation “1 + 0 = 1”. Similarly, as shown in the middle column of Figure 2a, when a skyrmion is generated only at input B, the input signal is represented as “0 + 1”. The magnetic skyrmion subsequently undergoes a similar process that realizes the logical operation “0 + 1 = 1”. In addition, the logical operation “1 + 1 = 1” is depicted in the right column of Figure 2a, where skyrmions are generated at both input A and input B, corresponding to the input signal “1 + 1”. The two skyrmions traverse a narrow nano-racetrack, merge into a magnetic domain wall, and finally reconvert into a magnetic skyrmion at the output, thereby accomplishing the logical operation “1 + 1 = 1”.

Figure 2b shows the operation of the logic AND gate, whose basic operations are defined as “0 ∙ 0 = 0”, “1 ∙ 0 = 0”, “0 ∙ 1 = 0”, and “1 ∙ 1 = 1”. The primary distinction between the logic AND gate and the logic OR gate lies in the increased width of the central nano-racetrack. This design ensures that a single skyrmion cannot reach the output, unless two skyrmions merge and transform into a larger domain wall. For example, as shown in the left and middle columns of Figure 2b, when only one skyrmion is generated at the A or input B, the input signal corresponds to “1 ∙ 0” or “0 ∙ 1”. In this case, the domain wall formed by the individual skyrmion is unable to occupy the entire width of the widened nano-racetrack and eventually annihilates at the upper right or lower right corner. Consequently, no magnetic skyrmion signal is detected at the output, thus successfully achieving the logic operations “1 ∙ 0 = 0” and “0 ∙ 1 = 0”. Whereas, when both input A and input B create a skyrmion, the input signal at this time is “1 ∙ 1”. In this scenario, the two magnetic skyrmions merge to form a domain wall spanning the full width of the wide nano-racetrack, and then subsequently transform back into a magnetic skyrmion, realizing the logical operation “1 ∙ 1 = 1”.

It should be noted that the design of these logic gates, like that of most current skyrmion-based logic gates, relies on integrating logical functions within specific nano-racetrack geometries (as shown in Figure 2). This geometric dependence poses a fundamental challenge for building large-scale, programmable integrated devices from uniform building blocks. To address this limitation, researchers have proposed more versatile integrated designs, in which a single racetrack structure can implement multiple logic functions [77,141,174,175,176,177,178,179,180,181,182,183,184].

**Figure 3:**
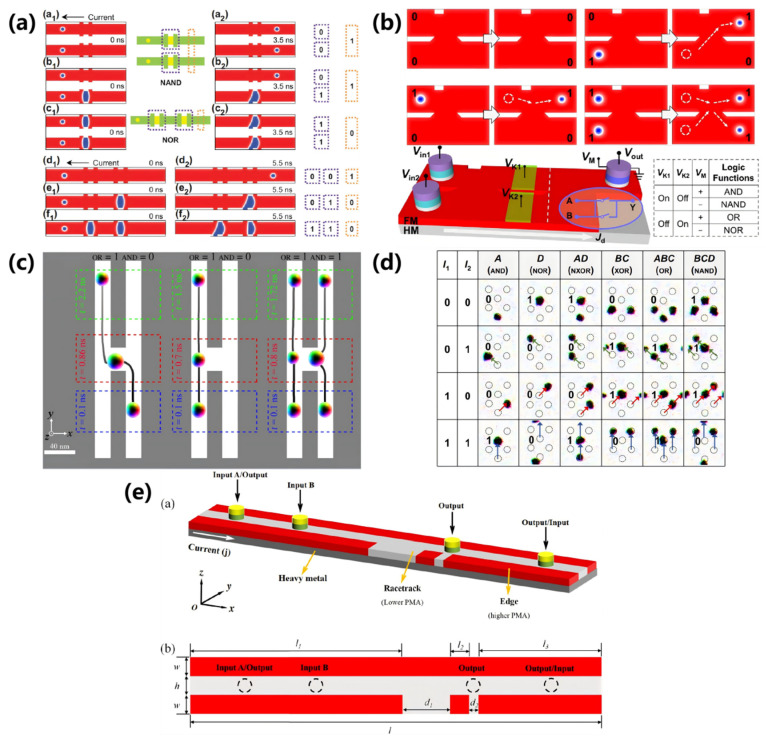
Different schematic diagrams for implementing magnetic skyrmion logic gates. (**a**) Logic gates based on the collision of magnetic skyrmions and domain walls [174]; (**b**) Reconfigurable logic gate [176]; (**c**) Reversible logic gates [178]; (**d**) Programmable logic gates [182]; (**e**) Schematic diagram of the racetrack design that integrates logical operations with diode functions [183].

For example, as shown in Figure 3, Xing et al. achieve the functionalities of logic NOT, NAND, and NOR gates through collisions between magnetic skyrmions and domain walls [174]. In these approaches, the presence or absence of domain walls represents the logical “1” and “0” of the input signal, while the presence or absence of skyrmions indicates the output signal “1” and “0”. Luo et al. control the “ON” and “OFF” states of a voltage-controlled barrier to direct the motion of magnetic skyrmions, thereby realizing the logic AND and logic OR gates [176]. Notably, they proposed that the magnetic moment direction of the fixed layer in the magnetic tunnel junction (MTJ) could be manipulated by applying positive and negative voltages [176]. This plan enables the conversion between the logic AND and NAND gates, as well as between the logic OR gates and NOR gates. Chauwin et al. exploit the SkHE to simultaneously implement reversible logic AND and OR gates within an H-shaped nano-racetrack [178]. Moreover, Yan et al. employed the “ON” and “OFF” states of an electric current to represent the input signals “1” and “0”, respectively, and utilized the pinning effect of skyrmions to implement programmable full-function logic gates [182]. Additionally, Shu et al. utilize both the SkHE and boundary effects to integrate the logic gate and diode functions in a single nano-racetrack [183,184].

In some logic architectures, the controlled conversion between skyrmions and magnetic domain walls is used to implement specific functions, such as generating skyrmions and domain walls at fixed positions for logic operations [174]. This strategy aims to combine the advantages of skyrmions as low-energy, stable information carriers with the ease of generating and controlling magnetic domain walls in local regions. However, while the hybrid approach facilitates the initialization or capture of skyrmions in designated geometries, its conversion process is often constrained by challenges in energy efficiency, speed, and stochasticity, ultimately limiting its scalability in complex, cascaded circuits. Consequently, current research increasingly focuses on all-skyrmion schemes, which are designed to establish a more compact and scalable spin-computing architecture through efficient and deterministic manipulation of skyrmions.

## 3. The Logic Gates in Synthetic Antiferromagnetic Materials

The SkHE in FM systems causes the trajectories of skyrmions to deviate from the direction of the driving current [186,187,188]. This phenomenon occurs because skyrmions in FM systems possess nonzero topological charges, resulting in a Magnus force perpendicular to the current direction. Consequently, the skyrmion deviates from straight propagation, which constitutes the skyrmion Hall effect (SkHE). Fundamentally, this behavior stems from the interplay between the skyrmions’ topological structures and spin–orbit coupling [73]. This transverse motion makes them susceptible to annihilation at sample boundaries, leading to information loss, which is a critical concern for memory storage applications. Significant research efforts have been devoted to eliminating or mitigating the SkHE [189,190,191,192,193].

In this context, SAFM skyrmions, comprising a pair of sublattice FM skyrmions with opposite topological charges that are tightly bound by AFM exchange coupling, have been proposed as a viable solution [73]. In SAFM skyrmions, the Magnus forces acting on the two sublattices are directed oppositely yet remain nearly equivalent in magnitude, resulting in their macroscopic cancelation and achieving quasi-linear motion [73]. This mechanism not only addresses the boundary annihilation challenge but also simplifies trajectory prediction and control within the circuit. By effectively mitigating issues associated with the Skyrmion Hall effect, SAFM skyrmions have catalyzed the development of innovative spintronic devices [77,159,163,169,177,181].

**Figure 4:**
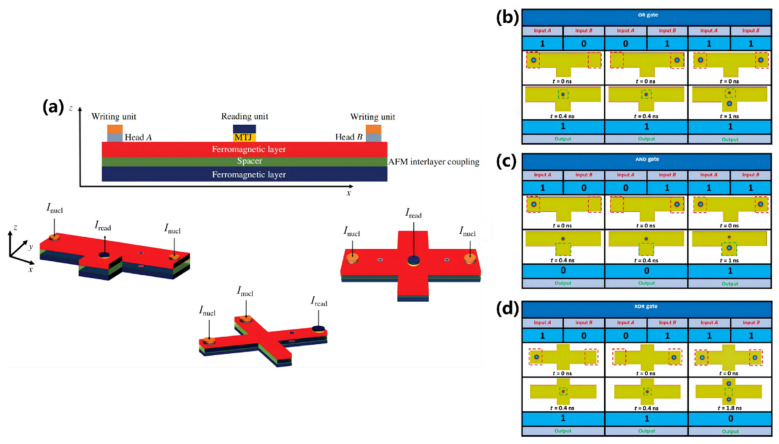
Logic gates based on SAFM skyrmions [181]. (**a**) Schematic diagram of the racetrack structure design; (**b**) Operating principle of logic OR gate; (**c**) Operating principle of logic AND gate; (**d**) Operating principle of logic XOR gate.

Based on the physical characteristics of SAFM skyrmions without SkHE, Fattouhi et al. design racetrack structures, as illustrated in Figure 4 [181]. The SAFM structure comprises two FM layers with anti-parallel magnetic moments, separated by a non-magnetic coupling layer. In this design, logic functions are realized through a combination of controllable current-driven motion, repulsive magnetic dipole interactions between skyrmions, and potential barrier interactions at the racetrack boundaries [181]. Figure 4a depicts the overall structure of the SAFM skyrmion-based logic gates, and Figure 4b–d present the schematic diagrams of each logical operation, respectively. In these diagrams, the red dashed boxes on both sides denote the inputs, and the green dashed boxes in the center represent the outputs. Electric currents flow from both sides to the center, causing the skyrmions at the inputs to migrate inward.

In the logical OR operation shown in Figure 4b, if only one skyrmion is generated at either the left or right input, it moves linearly under current drive to the center of the racetrack and is detected as an output, thereby achieving the logical functions “1 + 0 = 1”, or “0 + 1 = 1”. When skyrmions are generated at both inputs simultaneously (i.e., an input signal of “1 + 1”), both skyrmions move toward the center. The combined effects of the applied currents, boundary interactions, and skyrmion-skyrmion repulsion eventually force the skyrmions to a stable position. That is, one skyrmion is positioned near the upper boundary and the other near the lower boundary, as shown in the right column of Figure 4b. At this point, only the upper skyrmion is detected at the output, implementing logical operations “1 + 1 = 1”. Figure 4c shows the operation of the logic AND gate. Although its operational principle is similar to that of the OR gate, the primary distinction lies in the output position; the AND gate’s output is located lower. Consequently, a signal is detected only if both skyrmions converge at the center, allowing the lower skyrmion to be read. This condition implements the logical AND function “1 ∙ 1 = 1”, while the other two cases correspond to “1 ∙ 0 = 0” and “0 ∙ 1 = 0. Additionally, Fattouhi et al. design and implement a logic XOR gate, as shown in Figure 4d, with its output located at the geometric center. In the cross-shaped racetrack configuration, when two skyrmions are simultaneously input, they repel each other at the center, resulting in an output of “0” (i.e., “1 ⊕ 1 = 0”), as shown in the right column of Figure 4d. Conversely, when only one skyrmion is input, it is successfully detected, corresponding to the “0 ⊕ 1 = 1” and “1 ⊕ 0 = 1” logic cases.

**Figure 5:**
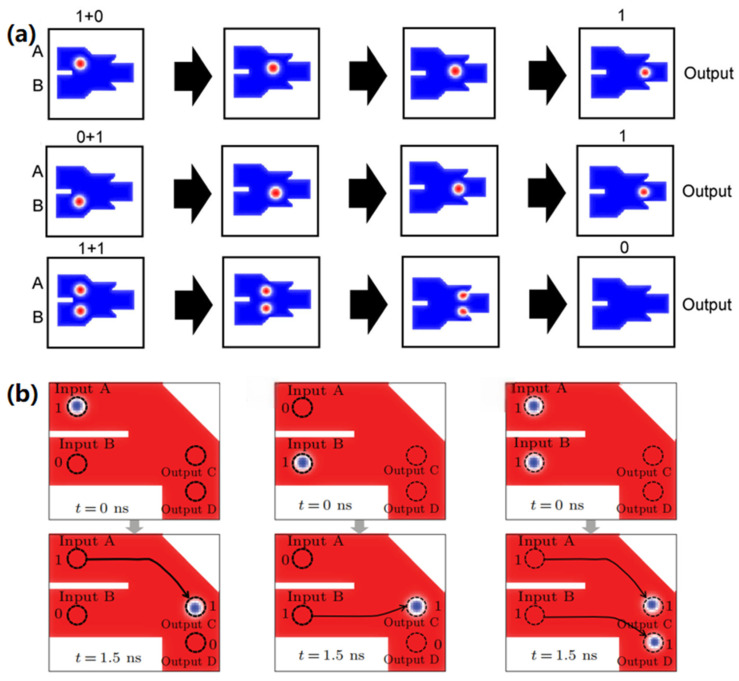
Logic operations based on SAFM skyrmions. (**a**) Logic XOR gates [177]; (**b**) Logic AND and OR gates [77].

The potential of these physical mechanisms is further demonstrated in more complex computing schemes. As depicted in Figure 5, Mak et al. and Li et al. employ distinct design strategies. Specifically, Mak et al. demonstrate half-adders and full-adders as well as a logic XOR gate via the design of specialized racetracks [177]. Figure 5a illustrates the operational process of the logic XOR gate. While Li et al. implement logic AND, OR, NOR, XOR, NOT, and NAND gates by leveraging boundary effects and skyrmion-skyrmion repulsion [77]. Figure 5b illustrates the operational process of the logic AND and OR gates, where the two logic gates also ingeniously utilize differences in their output positions to achieve distinct logical functions.

## 4. The Logic Gates in Antiferromagnetic Materials

AFM skyrmions, characterized by a zero topological number, are also inherently immune to the SkHE [83,85,86]. Recent studies further indicate that, under identical external conditions, AFM skyrmions exhibit higher longitudinal velocities compared to both FM and SAFM skyrmions [85]. Consequently, these attributes position AFM skyrmions as a highly promising derivative of FM skyrmions. Nonetheless, owing to their null net magnetic moment, the experimental detection of AFM skyrmions remains challenging [194,195].

**Figure 6:**
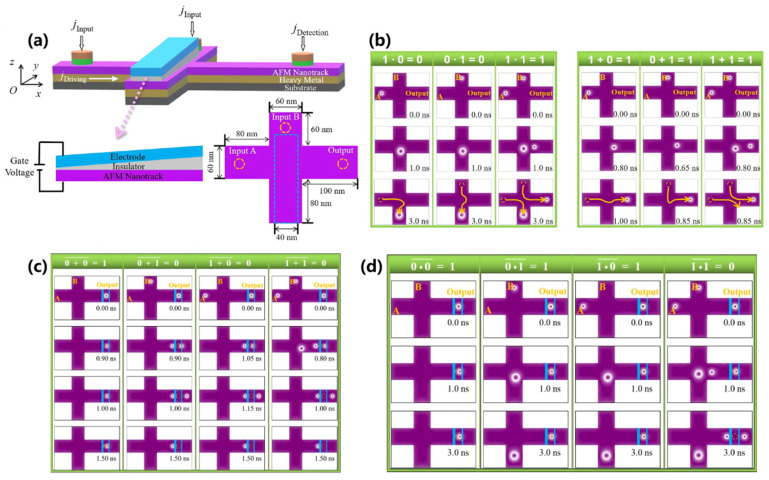
Logic gates based on AFM skyrmions [141]. (**a**) Three-dimensional illustration of the AFM skyrmion-based logic AND/OR gates; (**b**) Micromagnetic simulations of logic operations for the AND and OR gates, with skyrmion trajectories depicted by yellow lines and initial skyrmion positions marked by dashed circles; (**c**) Demonstration of the logic NOR gate; (**d**) Demonstration of the logic NAND gate.

The design and operational principles of AFM skyrmion-based logic gates are depicted in Figure 6 [141]. Liang et al. employ a cross-shaped AFM nanotrack wherein the motion of the skyrmions is jointly governed by an electric current and a magnetic anisotropy gradient [141]. In the model presented in Figure 6a, two inputs (A and B) and one output (C) are configured. A spin-polarized current is applied along the longitudinal nanotrack, whereas the transverse branch features a voltage-controlled magnetic anisotropy (VCMA) gradient in the blue region but no driving current. As illustrated in the left panel of Figure 6b, if only one AFM skyrmion is generated at input A, it will be driven by the spin current toward the intersection of the nanotrack. When the driving current density is below a critical threshold, the AFM skyrmion is directed to the lower terminal of the transverse branch under the influence of the VCMA gradient. Conversely, if the driving current density exceeds the critical value, the AFM skyrmion reaches output C along the longitudinal branch, as shown in the right panel of Figure 6b. Consequently, the logic operations for an AND gate (“1 ∙ 0 = 0”) and an OR gate (“1 + 0 = 1”) can be successfully implemented. Similarly, if an AFM skyrmion is generated exclusively at input B, it will either move downward to the lower terminal at a lower driving current density, or proceed to output C at a higher driving current density, thereby enabling the logical operations for an AND gate (“0 ∙ 1 = 0”) and an OR gate (“0 + 1 = 1”).

When two AFM skyrmions are generated at inputs A and B, they arrive at the intersection region nearly simultaneously, where their subsequent dynamics are governed by the driving current density. Under lower driving current densities, the AFM skyrmion from input A displaces the one from input B toward the longitudinal output C before proceeding along the lower transverse nanotrack. Conversely, at higher driving current densities, both skyrmions initially move longitudinally; however, the AFM skyrmion from input A is subsequently repelled by the one from input B and annihilates at the lower-right corner, resulting in only the AFM skyrmion from input B reaching output C. Thus, the logic operations corresponding to an AND gate (“1 ∙ 1 = 1”) and an OR gate (“1 + 1 = 1”) can be implemented. The trajectories of the AFM skyrmions, delineated by yellow lines, are illustrated in Figure 6b. Furthermore, by integrating the logic gates shown in Figure 6b, additional gates can be realized. In Figure 6c,d, when the incoming skyrmions approach the output, they are obstructed by the left voltage-controlled barrier. Concurrently, these incoming AFM skyrmions repel the resident AFM skyrmions at the output, causing the output signal to be “0”. Therefore, the NOR and NAND gates can be achieved. Finally, Liang et al. present a phase diagram for the practical application of logic AND and OR gate operations based on Micromagnetic simulations conducted under varied driving current densities and VCMA gradients.

## 5. Comparison Between Skyrmion-Based Logic Gates and Traditional CMOS Logic Gates

Although progress has been made in validating the operational principles of magnetic skyrmion-based logic gates, it remains necessary to evaluate their potential as candidates for future computing technologies through systematic comparisons with conventional CMOS technology. This section examines the performance and prospective advantages of skyrmion-based logic by focusing on critical dimensions such as computing speed, device size, energy efficiency, and computational paradigms.

Early studies were limited by driving current density and thermal stability, with skyrmion manipulation typically operating at the microsecond timescale. However, recent works based on SAFM and ferrimagnetic skyrmions have significantly altered this paradigm. For instance, implementing a full adder logic function in a SAFM racetrack system has reduced the overall delay to the nanosecond range (approximately 1.95 ns) [177]. More notably, in ferrimagnetic alloys such as CoGd, skyrmions can be reliably driven at speeds exceeding 0.83 km/s under current stimulation [196]. For a typical logic gate with a length of 50 nm, the intrinsic transmission delay is only about 50 picoseconds [196], suggesting that its theoretical switching frequency could rival or even exceed that of current mainstream CMOS processes.

Moreover, skyrmions can be as small as several tens of nanometers or even less than ten nanometers, endowing them with the intrinsic potential to serve as high-density information carriers. However, the implementation of complete logical functions necessitates additional structures, such as current channels and detection units, which often result in an overall footprint larger than that of equivalent CMOS circuits. Future optimization strategies should focus on developing all-electric control and detection schemes and integrating them with silicon-based processes. Notably, AFM or SAFM skyrmions, due to their nearly zero net magnetization, can effectively mitigate magnetic dipole crosstalk and exhibit distinct advantages in high-density integration schemes such as three-dimensional stacking [132].

Energy efficiency is also a critical focus in skyrmion logic and CMOS technologies. The driving current density for skyrmions can be as low as 10^5^–10^6^ A/cm^2^ [57]. Although the current density is comparable to that of CMOS logic, which employs charge (voltage) as a volatile information carrier, skyrmion logic leverages stable magnetic skyrmion topological structures that inherently provide non-volatility. The low operational energy consumption is achieved by directly driving the flipping or movement of these magnetic structures using spin current rather than charge current, thereby avoiding the significant Joule heat losses associated with CMOS circuits that rely on charge transport. Additionally, a key advantage of skyrmion logic lies in its non-volatile information storage; skyrmion logic gates do not require a continuous power supply to maintain their state during inactive periods, effectively eliminating the static power consumption related to leakage current in CMOS technology. Yagan et al. systematically analyzed the energy consumption composition in SAFM and FM skyrmion-based logic gates [197]. Their calculations partitioned the total energy consumption into two components: the change in magnetic energy, which corresponds to the energy required to flip the magnetic moment (SAFM: 0.00708–0.0125 fJ; FM: 0.0019–0.00517 fJ), and the energy associated with Joule heating from the driving current (SAFM: ~10–20 fJ; FM: ~130–270 fJ) [197]. It is important to emphasize that Joule heating constitutes the predominant fraction of the total operational energy consumption. For instance, in the case of an efficient SAFM skyrmion logic gate, the Joule heat energy per operation (~10–20 fJ) is approximately one to two orders of magnitude lower than that of intrinsic switching energy in advanced CMOS transistors (approximately 0.1–1 pJ, or 100–1000 fJ). Moreover, the inherent non-volatile characteristic of skyrmion logic, which results in zero static power consumption, establishes a robust physical foundation for the development of ultra-low-power systems.

One more important aspect is that the potential of skyrmion logic may catalyze a paradigm shift in computing. Its intrinsic non-volatility blurs the conventional boundaries between storage and computation, thereby naturally supporting integrated computing-storage architectures. Consequently, data transfer between the processor and memory could be significantly reduced, which may alleviate the well-known “memory wall” problem [132]. Furthermore, the collective dynamics, nonlinear responses, and other unique attributes of skyrmions position them as a promising physical platform for implementing neuromorphic, stochastic, and other post-Von Neumann computing paradigms [164,165].

Reading magnetic skyrmions is a pivotal step in device implementation. Early approaches relied on the inherent topological Hall effect of skyrmions, although the resulting signal was weak [198]. Currently, the predominant research focus is on employing magnetic tunnel junctions to detect substantial tunneling magnetoresistance changes caused by skyrmions [69]. These junctions offer strong signals and excellent technical compatibility, thereby overcoming previous obstacles to reliable detection. As a result, research has shifted toward optimizing and integrating these existing solutions to develop highly robust devices.

Furthermore, it is noteworthy that compared to the electric current as the driving mechanism, spin waves (magnons) offer a low-energy consumption alternative, with nearly zero Joule heating, and, as a spin excitation, they can engage in strong nonlinear interactions with skyrmions (e.g., scattering, modulation and excitation). Consequently, spin waves can directly serve as the information transmission medium between logic units [117,199,200], enabling the computation, storage and transfer of information entirely within the spin domain. By integrating the non-volatile storage and processing capabilities of skyrmions with spin waves, the “skyrmion-spin wave” hybrid architecture holds promise for addressing the challenges of conventional electronics related to power consumption, integration density, and non-volatility, thereby offering a potential pathway toward the development of highly energy-efficient and scalable all-spin computing systems.

## 6. Summary and Outlook

As conventional complementary metal-oxide-semiconductor technology nears its fundamental physical limits, the development of novel low-power, non-volatile computing architectures has become increasingly imperative. Magnetic skyrmions, characterized by their topological stability, nanoscale dimensions, and ultra-low driving current density, have emerged as promising candidates for next-generation high-speed, low-power spintronic devices. Serving as an innovative information carrier, magnetic skyrmions convey data through their controlled creation and dynamic manipulation, thus offering potential to surpass traditional charge-based logic gates. Based on the operating mode of the information carrier, existing magnetic skyrmion logic gate schemes can be divided into two categories: the hybrid scheme, which relies on the conversion between domain walls and skyrmions, and the all-skyrmion scheme, wherein the entire process, including generation, transmission, interaction, and readout, is conducted solely within the skyrmion state. Although the hybrid scheme is advantageous for certain initialization tasks, its conversion process is inherently limited in terms of energy efficiency, speed, and determinism, thereby restricting its scalability in circuit applications. As a result, research has increasingly focused on the all-skyrmion scheme, which promises to facilitate more compact and scalable spin-based computing architectures through the efficient and deterministic manipulation of skyrmions alone.

This review succinctly introduces the operating principles and recent advancements in logic gate devices based on FM, SAFM, and AFM skyrmions. Research demonstrates that distinct types of skyrmions exhibit unique characteristics: Ferromagnetic skyrmions are relatively easier to detect, albeit with velocity constraints imposed by the skyrmion Hall effect; Antiferromagnetic skyrmions display ultra-fast dynamic properties while posing significant detection challenges; and SAFM skyrmions provide a favorable balance between stability and speed. It is crucial to emphasize that magnetic skyrmion logic gates are not intended to entirely replace mature silicon-based technologies but rather to complement and extend them. Looking forward, the field faces numerous challenges and opportunities. On the one hand, breakthroughs in material systems, device fabrication, and integration processes are required to overcome key technical hurdles such as controlled skyrmion generation, precise detection, and stable motion. On the other hand, a synergistic integration of skyrmion-based logic with traditional circuits is essential to harness the unique advantages of both paradigms. Although further breakthroughs in control, generation and detection are necessary for skyrmion logic, the principles and solutions reviewed in this article have established a preliminary foundation for its development. Furthermore, beyond spintronic applications, skyrmions hold significant promise for neuromorphic and quantum computing. The integration of magnetic skyrmion-based technologies with traditional computing methods is anticipated to foster the development of diverse and high-efficiency computing architectures in the future.

It is noteworthy that in recent years, machine learning (ML) has emerged as a potent data-driven tool with significant potential to accelerate the design and optimization of skyrmion-based devices. For instance, for AFM skyrmions, neural network models have been effectively trained to predict the stable formation regions under various conditions, achieving a prediction accuracy exceeding 97% [201]. Similarly, active learning frameworks combined with Gaussian process regression have been employed to efficiently map the skyrmion phase diagrams in two-dimensional magnets, thereby elucidating precise quantitative relationships between phase boundaries and critical magnetic parameters [202]. These studies demonstrate that ML not only serves as an efficient screening tool but also provides a novel perspective for deeply understanding the stabilizing physical mechanisms of skyrmions by parsing multi-parameter competition. Integrating ML into the design workflow of skyrmion logic devices holds the promise of enabling acceleration of the entire workflow, from material screening and parameter optimization to performance prediction, potentially serving as a key enabling approach to propel this technology toward practical application.

## Data Availability

No new data were created or analyzed in this study.
